# Supplementary material for: Beyond the Heart: The Predictive Role of Coronary Artery Calcium Scoring in Non-Cardiovascular Disease Risk Stratification
Source: Diagnostics (Basel). 2024 Oct 22;14(21):2349. doi: 10.3390/diagnostics14212349 (PMC11545064; doi:10.3390/diagnostics14212349)
Supplement: Supplementary file 1 [file diagnostics-14-02349-s001.zip › diagnostics-3247377-supplementary.pdf]

Disease entity	Study	Study Design	Uses of CT Calcium Scoring	Population under study	Implications and results	Limitations and additional comments	PMID/doi
Diabetes Mellitus	1) Jingchuan Guo, 2019	Prospective cohort study	Risk prediction of incident CAD in type 1 diabetics	292 type 1 diabetic patients, no CAD, followed up for 10.7 years.	People with type 1 diabetes with CACS>100 are twice as likely to have incident CAD than those with CACS<100	Incorporating CAC with other risk models can improve risk prediction in Type 1 Diabetes	PMID: 30426170
	2)Harold S Starkman, 2003	Cross-sectional study	CAC is used to estimate the prevalence of early CAD in young patients with type 1 DM.	101 type 1 diabetics with more than five years duration, aged 17-28 years, no history of CAD	-Smoking and Lp(a) are risk factors for elevated CAC in type 1 DM patients.<br>-High CAC is associated with early CAD in young patients with type 1 DM	The cohort size was small, and hence, the power of the study is limited.	PMID: 12547875
	3) Erica P Gunderson, 2021 The CARDIA study	Prospective cohort study	Worsening glucose tolerance is seen in women with a history of GDM and having elevated CACS.	1133 women without diabetes at baseline, ≥1 birth at follow-up	Women with a history of GDM were twice as likely to develop CAC (score ≥ 100) within 15 years after pregnancy, irrespective of	Normoglycemic status in patients with a history of GDM does not decrease the atherosclerotic risk.	PMID: 33517667

					their glucose status.		
	4)Miodrag Dikic, 2015	Prospective cohort study	Prognostic role of CACS in asymptomatic diabetic patients	200 asymptomatic patients; 101 with DM	CAC is a vital prognostic tool for diabetic patients with a coronary calcium score (CCS) ≥200, indicating unfavorable outcomes	CAC is an independent predictor of poor outcomes in DM	PMID: 26340922
	5)Valentina Valenti, 2016	Prospective cohort study	CACS used in the prognosis of asymptomatic DM patients	9715 patients with 14.7-year follow-up	CACS=0 indicates favorable prognosis for five years in asymptomatic DM patients	- Inability to assess cause-specific mortality<br>-Single center design is another limitation	PMID: 26848062
	6) Shaista Malik, 2017 Multi-Ethnic Study of Atherosclerosis	Prospective cohort study	CAC for long-term risk classification in Type 2 DM	6814 participants, 45 to 84 years without known CVD, 4 race/ethnicity groups	A CAC score of 0, regardless of the length of diabetes, use of insulin, or glycaemic management, is linked to a lower risk of CVD	-lack of data on diabetes duration for the entire cohort<br>-limitation in witnessing the effects of CAC progression	PMID: 29117273
	7) Bahram Khazai, 2021 Multi-Ethnic Study of	Prospective cohort study	CAC as a screening tool for CVD in diabetics with retinopathy	6814 participants, 45 to 84 years without known CVD, 4 race/ethnicit	significant link between retinopathy and elevated CAC in DM patients but not in non-	-differences in retinal photography techniques<br>-higher missing data in the DM group<br>-lack of analysis	10.1016/j.amjcard.2021.03.026.

	Atherosclerosis			y groups	DM individuals	on new retinopathy in those with CAC progression.	
Primary Hyperparathyroidism	1) Streeten, E.A, 2008<br><br>2)Mesquta, P.N, 2017<br><br>3) Kepez, A, 2009	Case-control study	The utility of CAC scoring still needs to be tapped.	The study compared Participants with pHTP with age and gender-matched controls.	Although evidence in animal models is motivating, human studies have yet to show favorable results, given the small sample size and study limitations. This reflects the need for broader trials.	-These studies have a small sample size. The generalisability of this study is thus questionable.	1) PMID: 18308652<br><br>2) PMID: 28790836<br><br>3) PMID: 18784985
Sex Hormones	1) Shores, M.M, 2014	Longitudinal cohort study	Not used	1032 men from the Cardiovascular Health Study (CHS) free of CVD at baseline	-The study found no relation between Total and free testosterone levels and incident cardiovascular disease	-The study utilized a single Testosterone value in contrast to guideline-recommended serial hormone level measurement.<br>-The day of blood collection was not standardized.	PMID: 24628549
	2) Meyer, E, 2018	Review article	Not used	Review article	The study discusses the heterogeneous results of studies evaluating the association between serum testosterone levels and	-Variations in the study designs and population demographics.	PMID: 29384142

					CVD.		
	3) Khazai, B, 2016	Multicenter study	CAC scores were seen to be associated with low testosterone levels.`	3164 men without known CVD in the MESA cohort	The study found that lower free testosterone levels were associated with high CAC scores and low total testosterone levels were associated with high log CAC scores.	High log CAC scores were linked to lower testosterone, while lower carotid intima-media thickness was associated with lower total testosterone levels.	PMID: 26663365
	4) Subramanya, V, 2019	Multicenter study	Monitoring CAC progression can be used to assess CVD risk in post-menopausal females.	2759 post-menopausal women	The study observed that women with high baseline free testosterone levels had more prevalent coronary artery calcification (CAC) at follow-up, with significantly greater progression in CAC.<br>-Thus, this subset of populations can benefit from earlier CVD risk-reducing strategies.	The study needs to capture the impact of perimenopausal hormonal changes.<br>-The analysis is also subject to survival bias.	PMID: 30297127
Primary Hyperaldosteron	1) Inoue, K, 2020	Multicenter study	CAC scoring can predict	948 adults from the	Higher CAC scores are	The study did not take into account	PMID: 32418495

ism			poor outcomes in patients with elevated serum aldosterone levels	MESA cohort	associated with increasing serum aldosterone levels, especially with suppressed renin phenotype. Patients with suppressed renin phenotype had a higher all-cause mortality rate.	various unmeasured confounders. A single measurement of serum aldosterone level limits the study's utility to establish a temporal relationship.	
	2)Monticone, S, 2018	Meta-analysis and multicenter Study	Not used	Thirty-one studies, including 3838 patients with primary aldosteronism and 9284 patients with essential hypertension, were included.	The meta-analysis showed a statistically significant increased risk of CVD and non-CV diseases like DM and metabolic syndrome in patients with primary hyperaldosteronism as compared to those with essential hypertension.	The review suggests a possible independent association of aldosterone with vascular calcification.	PMID: 29129575
Osteoporosis	1) Rui Xu, 2016	Cross-Sectional study	Association between Bone Mineral	186 postmenopausal women, 50–	T-scores are independent	BMD of spine and femoral neck was measured using DEXA,	PMID: 27363000

			Density and CAC	80 years of age	predictors of CAC scores	which proved to be a marker for atherosclerosis	
	2) N Ahmadi, 2018	Observational Cohort study	Elevated CACS correlated with increased all-cause mortality	5590 at-risk subjects, age 57 ± 12, 69% male, no previous CAD	Lower BMD is linked to a higher CACS >0	Investigated both genders across ethnicities	PMID: 29704026
	3) Catherine E Handy, 2016 Multi-Ethnic Study of Atherosclerosis	Prospective cohort study	CAC score helps in risk prediction of hip fractures	6814 participants followed for 10.2 years	-Doubling CAC scores increased hip fracture risk by 10%.<br>-CAC=0 reduces hip fracture risk by 69%	-The study might have missed patients treated only in outpatient setups.<br>-The cohort with hip fractures was small.	PMID: 26970999
	4) S N Lee, 2016	Cross-Sectional study	Association between CACS and osteoporosis noted	863 asymptomatic post-menopausal females	High CACS (≥100) were linked to osteoporosis, independent of CV risk factors and age.	A trend towards association with multivessel disease found	PMID: 27397609
	5) Josephine Therkildsen, 2021	Cross-sectional studies	Association between BMD and CACS varies between different gender	1487 participants (mean age 57 years; 47% men)	Lower BMD was present with increased CACS in women but not in men	Studies clarifying possible different pathophysiologies in both genders are needed.	PMID: 31668962
	6) Yaffah L Wiegandt, 2019	Cross-sectional studies	Association between BMD and CACS varies between	1163 men and 1385 women, 61 ± 10 years	An inverse relation between CACS and	The role of estrogen needs to be clarified, and disparities in results between	PMID: 30659977

	Wiegandt's (Copenhagen General Population Study)		different gender		BMD in men and post-menopausal female but not premenopausal female	genders need further investigation.	
Dementia	1)Fujiyoshi, A 2017	Prospective Cohort Study	CAC scores can predict poor outcomes in patients with dementia.	6293 participants of the MESA cohort, free of cardiovascular disease and normal baseline cognitive function.	The study found a statistically significant trend (P=0.026) of increasing risk of dementia with higher baseline CAC values	-unable to demonstrate the standalone contribution of CAC in cerebral small vessel disease<br>-The observational nature of the study limits	PMID:-28465455
	2)Bos, D 2012	Cross-sectional study	CAC scores can correlate with brain volumes and the likelihood of neurodegenerative diseases.	2414 non-demented participants of the Rotterdam study	Higher calcification correlated with lower cognitive scores, especially for aortic arch calcification	-A single cognitive assessment limited the evaluation of cognitive decline over time.<br>-It used two types of MDCT scans.	PMID: 22537801
	3) Vidal, J.-S, 2010	Cross-sectional study	CAC scores can predict cognitive decline.	4085 non-demented participants of the AGES-Reykjavik Study	Higher CAC scores were associated with reduced brain volumes and a significant cognitive decline	-the study mainly included elderly participants with a median age of 76.<br>-temporal association between coronary artery calcification and cognitive decline cannot be established.	Doi: 10.1161/STROKEAHA.110.579581

	4) Kuller, L.H, 2016	Prospective cohort study	CAC scores can predict the likelihood of cognitive decline in older people.	532 participants of the Cardiovascular Health Study-Cognition Study, mean age >80 years	Participants with zero and low CAC scores have a lower rate of cognitive decline.	-The study's cohort included only those who survived into their 80s, which may limit the generalizability to a broader population.	PMID: 26940919
	5) Ya-Nan Ma, 2021	Cross-sectional study	CAC is used to determine the prevalence of dementia risk	1332 participants, aged 40-80 years	The prevalence of high dementia risk increased with CAC: 24.79% (CACS ≥ 400).	CAC ≥ 400 has moderate accuracy in identifying high-risk dementia patients	PMID: 34404988
Cognitive Function and Psychosocial Wellbeing	1)Ya-Nan Ma, 2021	Cross-sectional study	Association between CAC and cognitive function & memory	1332 participants, aged 40-80 years, free of dementia in Beijing	CACS ≥ 400 is associated with greater cognitive decline	- unable to determine the predictive role of CAC in cognitive decline	PMID: 34404988
	2) Imke Janssen, 2022	Longitudinal cohort study	Association of Psychosocial Wellbeing and CAC Progression	312 Black and White women free of CAD (SWAN study)	psychosocial well-being predicts the progression of CAC	Assessment of well-being was self-reported	PMID: 35191325
Chronic Obstructive Pulmonary Disease	1)Catherine E Handy, 2016	Prospective cohort study	CAC score is used to predict new diagnoses of COPD	6814 participants from 6 Multi-Ethnic Study of Atherosclerosis	CAC score of >400 was associated with a 271% higher risk of COPD diagnosis, Doubling of CAC score led to a 10% heightened risk of COPD diagnosis.	New diagnoses were obtained via ICD hospital coding, so the time to diagnosis may not be same as time to disease onset	PMID: 26970999

	2)Allison W Peng, 2021	Prospective cohort study	CAC score is used to find its association with increased risk of COPD diagnosis	6814 participants from 6 Multi-Ethnic Study of Atherosclerosis	Subjects with a CAC score of >1000 are associated with an increased risk of COPD diagnosis than those with 400-999	Sample size of subjects with CAC score > 1000 was relatively few in number	PMID: 33650435
	3)Surya P Bhatt, 2018	Multicenter study	CAC score is used in predicting CVD in patients with COPD	1875 current and former smokers	Weston score of ≥ was associated with time to first acute coronary event.(hazard ratio= 2.16)	The CT scans were not electrocardiographically gated	PMID: 29890123
	4) Hye Yun Park, 2010	Retrospective cross sectional study	Comparing CAC score with lung function	4905 adult male patients of a health promotion center between March 2005 to February 2008	The ORs for the presence of coronary artery calcification in the lowest quartiles of FVC and FEV(1) (% pred) were 1.31 (95% CI, 1.09-1.58; p=0.004) and 1.22 (95% CI, 1.02-1.46; p=0.029) respectively.	The study was conducted in a single center	PMID:20335013
	5)Eun Jin Chae, 2013	Prospective cohort	Used to find the association between CAC score and the extent of emphysema	134 patients with COP and 101 smokers without airflow limitation	significant associations between coronary calcium score and forced expiratory volume at 1 second/forced	Lack of evaluation of factors other than smoking that influence lung function	PMID: 24045251

					vital capacity (P = 0.016)		
	6)Michelle C William, 2014	Multicenter study	Association of CAC score and lung functional capacity and outcome in patients with COPD	672 patients with COPD, 199 smokers with normal spirometry and 71 non-smokers	CACS was higher in patients with COPD than in smokers or non-smokers	Cardiovascular events were not recorded	PMID: 24473329
Pulmonary Embolism	1)Catherine E Handy, 2016	Prospective cohort study	Association of CAC score and diagnosing Pulmonary embolism	6814 participants from 6 Multi-Ethnic Study of Atherosclerosis	Did not find any association	New diagnoses were obtained via ICD hospital coding, so the time to diagnosis may not be same as time to disease onset	PMID: 26970999
	2)N vander Bijil, 2016	Case control study	Association between CAC score and unprovoked pulmonary embolism	100 patients with pulmonary embolism and 100 patients in whom pulmonary embolism was ruled out	No association was found	This study was limited by small sample size.	PMID: 26865284
	3)M C Williams, 2019	Retrospective cohort	Asses CAC score in patients with PE to correlate with mortality	400 participants with pulmonary embolism	No association found	Study suggest there could be selection bias as most patients with severe PE could die before reaching a hospital.	PMID: 31615632
Pneumonia	1)Catherine E Handy, 2016	Prospective cohort study	Asses CAC score with risk of developing	6814 participants from 6 Multi-	CAC>400 had a two-fold elevated risk	New diagnoses were obtained via ICD hospital coding, so the	PMID: 26970999

			pneumonia	Ethnic Study of Atheroscler osis	of developing pneumonia	time to diagnosis may not be same as time to disease onset	
	2)Tuichiro Takeshita,2 021	Retrospectiv e cohort	To determine the prognostic value of CAC score in COVID-19 patients	53 consecutive patients with COVID-19	CACS≥180 is associated with deteriorating oxygen levels and CT chest findings	Ct scans were non-ECG gated	PMID:34272 254
	3)Yogesh Sean Gupta, 2021	Retrospectiv e cohort	To evaluate the relationship between CAC scores and patient outcomes.	180 COVID-19 patients ≥ age 21 admitted from March 1, 2020 to April 27, 2020	Severe CAC score was independentl y associated with intubation and mortality	Suggests there could be observer bias in the CAC assessment	PMID: 33601125
	4)Francesco Giannini, 2021	Multicenter study	To evaluate the association of coronary artery calcium and total thoracic calcium on in-hospital mortality in COVID-19 patients.	1093 patients from 16 Italian hospitals with COVID-19 and an admission chest CT for pneumonia severity assessment were included	higher inpatient death rate among those with higher CAC scores(HR-1.308)	Study population included only COVID-19 patients who underwent chest CT for lung assessment	PMID: 33744175
Sarcoidosis	1)Mehmet Sait Altintas, 2021	Case control study	To assess CAC score and sarcoidosis association	45 controls and 78 sarcoidosis patients	No association found		PMID: 33390580

Chronic Kidney Disease	1) Bundy, J.D, 2018	Multicenter study	CAC scores are associated with markers of declining renal function	1123 CKD patients enrolled in the Chronic Renal Insufficiency Cohort (CRIC) study.	Declining glomerular filtration rate (GFR), elevated Cystatin C levels, PTH, and FGF-23 all lead to CAC progression by more than 100 Agatson units over a 3-year follow-up period.	The study had slightly differing definitions for CAC progression.	PMID: 29459266
	2)Wang, X,-R, 2019	Systematic review and meta-analysis	Higher CAC scores signify poor prognostic outcomes.	Meta-analysis of 38 cohort and cross-sectional studies.	Higher CAC scores are strongly associated with increased all-cause mortality.	The studies are heterogeneous, with varied demography, population sizes, and composition.	DOI: https://doi.org/10.1080/0886022X.2019.1595646
	3) Budoff, M.J, 2011	Cross-sectional study	CAC scores are indicative of declining GFR	1908 participants of the CRIC study	Higher CAC scores were associated with lower eGFR values	The study did not contain data on cardiovascular outcomes, thus limiting the information on the utility of CAC.	PMID: 21783289
	4) Bundy, J.D, 2019<br><br>5) Smith,E.R, 2014	Prospective cohort study	CAC scores reflect calcification propensity.	1274 participants from the CRIC study.	-Patients with higher calculated calcification propensity had more severe prevalent CAC and CAC progression	The study was unable to distinguish intimal from medial calcification.<br><br>Possibility of potential selection bias.	-DOI: 10.1053/j.ajkd.2019.01.024<br><br>-PMID: 24179171

					-Higher calcification propensity reflected increased incidence of all-cause mortality.		
	6) Chen, J, 2017	Prospective cohort study	CAC scores strongly predict poor cardiovascular outcomes in CKD.	1541 participants of the CRIC study without cardiovascular disease at baseline who had non-zero CAC.	Higher CAC scores were associated with more significant cardiovascular mortality in CKD.	-The study was unable to distinguish intimal from medial calcification.<br><br>-More clinical outcomes need to be achieved across CAC categories.	PMID: 28329057
Nephrolithiasis	Liu et al. (2023)	Cross-sectional, cohort study	Investigates the association between nephrolithiasis and CAC	Patients with recurrent kidney stones	Higher CAC scores in patients with kidney stones; 3x risk of severe coronary calcification (CAC > 400)	More studies are needed to clarify the association with coronary stenosis	https://doi.org/10.1007/s00345-023-04442-8.
Erectile Dysfunction	Osondu et al. (2018)	Systematic review, meta-analysis	Investigates CAC in patients with erectile dysfunction	Patients with erectile dysfunction, particularly younger men	ED may indicate subclinical CVD; could be used as a screening tool for CVD in younger me	Evidence inconclusive, more research needed	https://doi.org/10.1177/1358863X17725809

Metabolic dysfunction-associated steatotic liver disease (MASLD)	Lu et al. (2013)<br><br>Jaruvongvanich et al. (2016)<br><br>Kapuria et al. (2018)	Systematic review and meta-analysis<br><br>Systematic review and meta-analysis<br><br>Systematic review and meta-analysis	Not specifically focused on CT calcium scoring, but explores the overall link between NAFLD and CVD risk, which can be assessed with CT calcium scoring Investigates the association between NAFLD and coronary artery calcification using CT calcium scoring<br><br>Examines the association between hepatic steatosis (fatty liver) and subclinical atherosclerosis, with CT calcium scoring being one of the assessment tools	Patients with NAFLD across multiple studies<br><br>Patients with NAFLD across multiple studies<br><br>Patients with hepatic steatosis	NAFLD is independently associated with a higher risk of cardiovascular disease, indicating it is an important risk factor for CVD<br><br>NAFLD is significantly associated with higher levels of coronary artery calcification, indicating a higher risk for subclinical atherosclerosis<br><br>Hepatic steatosis is associated with a higher prevalence of subclinical atherosclerosis, suggesting the need for cardiovascular risk assessment in these patients	Limited by heterogeneity in study populations and diagnostic criteria for NAFLD and CVD<br><br>Limited by study designs and potential confounders not accounted for in individual studies<br><br>Variability in hepatic steatosis and subclinical atherosclerosis definitions across studies	24382937<br><br>27771411<br><br>30071762

Schizophrenia	Smith et al. (2007)<br><br>Fulda et al. (2022)	Cross-sectional study<br><br>Cross-sectional study	Examines coronary artery calcification (CAC) using CT calcium scoring in relation to hostile personality traits<br><br>Investigates the relationship between physical aggression and coronary artery calcification using CT calcium scoring	Middle-aged and older married couples<br><br>Participants from the North Texas Healthy Heart Study	Hostile personality traits, particularly when rated by spouses, were associated with higher levels of coronary artery calcification<br><br>Physical aggression was positively associated with coronary artery calcification, indicating a potential link between aggression and cardiovascular risk	Limited by reliance on self-reported personality traits and lack of longitudinal data<br><br>Limited by the cross-sectional design, which does not establish causality	17636153<br><br>34495391
Lung Cancer	Dzaye et al. (2021)	Large cohort study	Examines the link between CAC and lung cancer mortality	Over 55,000 participants	Strong correlation between high CAC (≥ 400) and lung cancer mortality, particularly in smokers	Current models for lung cancer prediction don't incorporate CAC data	https://doi.org/10.1016/j.atherosclerosis.2021.10.007.
Nonfunctioning adrenal incidentalomas	Akkus et al. (2021)	Cohort study	Assesses association between nonfunctioning adrenal incidentalomas and	Patients with nonfunctioning adrenal incidentalomas	NFAI group had significantly higher CAC scores, indicating higher	Limited data, more studies needed	https://doi.org/10.2174/1871530320666200910110337

			CAC		atherosclerosis risk		
Autoimmune diseases	Martínez-Ceballos et al. (2021)<br><br>Mendoza-Pinto et al. (2022)<br><br>Gartshteyn et al. (2019)	Systematic literature review and meta-analysis<br><br>Systematic literature review and meta-analysis<br><br>Cross-sectional study	Assesses coronary artery calcification (CAC) via CT calcium scoring in patients with various autoimmune diseases<br><br>Assesses asymptomatic coronary artery disease using CT calcium scoring in SLE patients<br><br>Examines coronary artery calcification prevalence using CT calcium scoring in young SLE patients	Patients with autoimmune diseases, including systemic lupus erythematosus (SLE), rheumatoid arthritis (RA), and others<br><br>Patients with systemic lupus erythematosus (SLE)<br><br>Young patients with SLE, predominantly of Hispanic and African-American descent	Coronary calcium scoring helps identify subclinical atherosclerosis in autoimmune disease patients, highlighting an elevated cardiovascular risk<br><br>Subclinical coronary artery disease is common in SLE patients; CT calcium scoring is useful for early detection<br><br>High prevalence of coronary artery calcification was found in this cohort, suggesting an elevated risk of early atherosclerosis	Limited by variability in study designs, potential heterogeneity of autoimmune conditions, and publication bias<br><br>Meta-analysis limited by small sample sizes and variability between included studies<br><br>Limited by small cohort size and lack of longitudinal data	35466098<br><br>35466098<br><br>31175129

Psoriasis	Wu et al. (1989)	Cross-sectional, meta-analysis	Assesses the link between severe psoriasis and CAC	Patients with severe plaque psoriasis	Significant association between severe psoriasis and higher CAC scores, particularly in younger patients	Molecular pathways are not fully understood, further studies required	https://doi.org/10.1136/bmj.298.6676.779.
Dental pathologies	1)H C M Donders, 2020	Retrospective cross-sectional study	Tooth loss is associated with higher CACS	212 patients from three hospitals across the Netherlands	More significant bone loss in those with higher CAC	The negative predictive value of CAC=0 for tooth loss is weak in symptomatic patients	PMID: 33326424
	2) Daniel W. Groves, 2015	Longitudinal cohort study	Periodontal disease is linked to long-term CAC progression	1021 participants from the CACTI study, out of which 473 had Type 1 DM	Periodontal disease in Type 1 diabetics is associated with CAC progression but not CAC prevalence	-the presence of periodontal disease was determined using a questionnaire instead of a clinical examination	PMID: 26189036
